# Supplementary material for: Susceptibility of lymnaeid snails to Fasciola hepatica and Fasciola gigantica (Digenea: Fasciolidae): a systematic review and meta-analysis
Source: PeerJ. 2025 Mar 14;13:e18976. doi: 10.7717/peerj.18976 (PMC11913016; doi:10.7717/peerj.18976)
Supplement: Supplemental Information 2 [file peerj-13-18976-s002.docx]

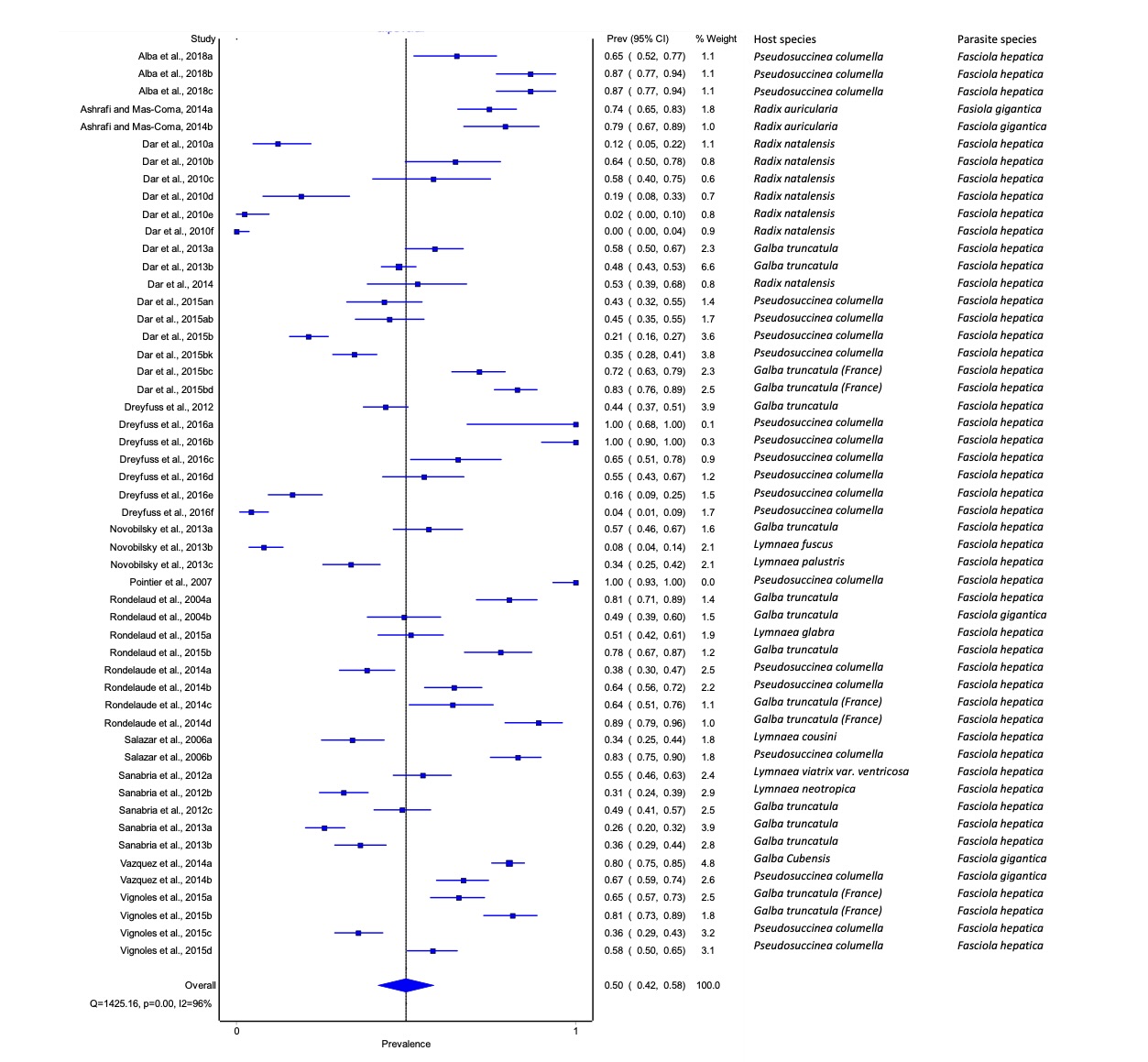


**Figure S1 Forest plot showing the overall experimental infection rates of *Fasciola hepatica* and *F. gigantica* in lymnaeid snails.**


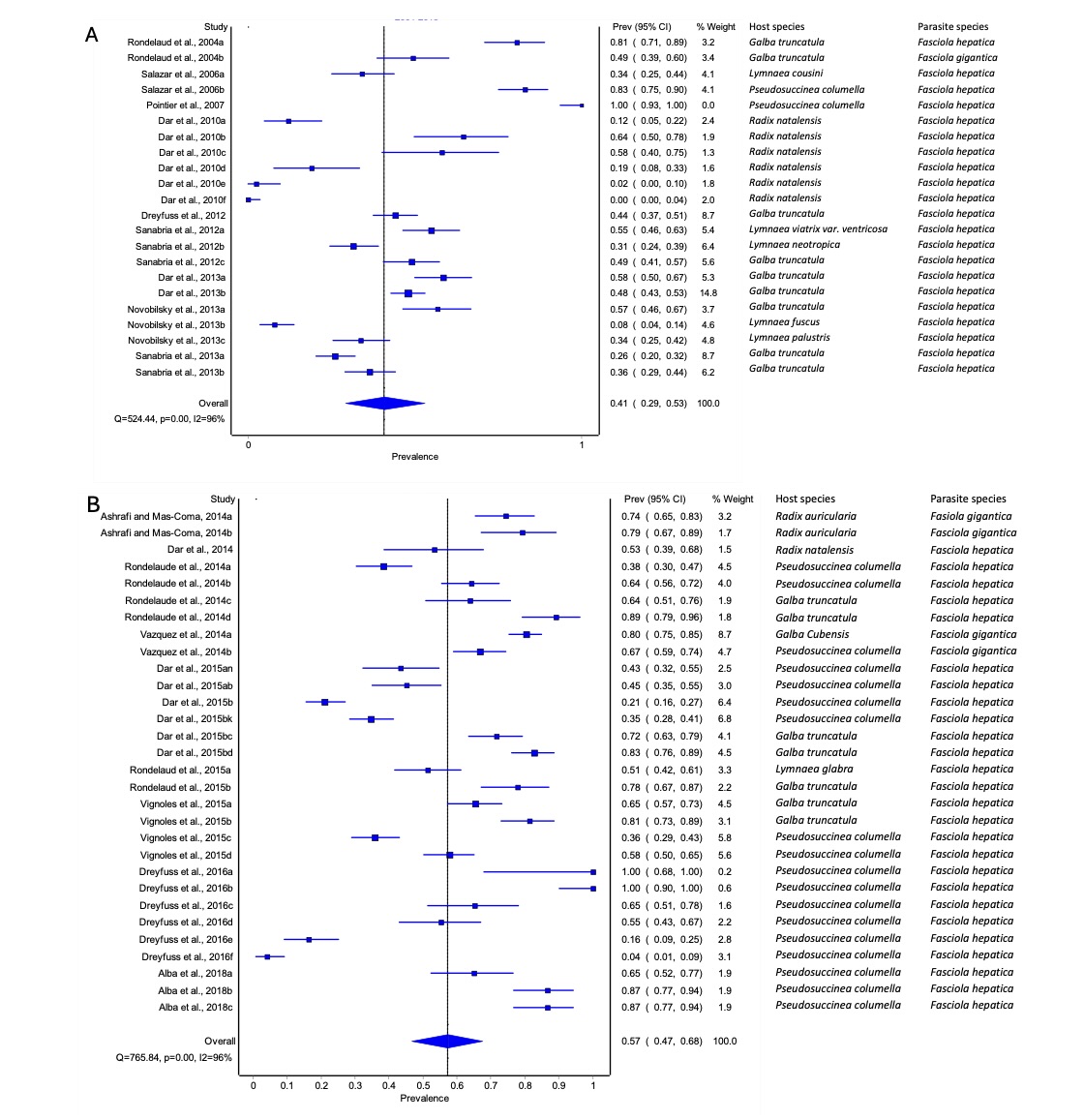


**Figure S2 Forest plots showing experimental infection rate of lymnaeid snails with *Fasciola* species from (A) 2004-2013 and (B) 2014-2023.**


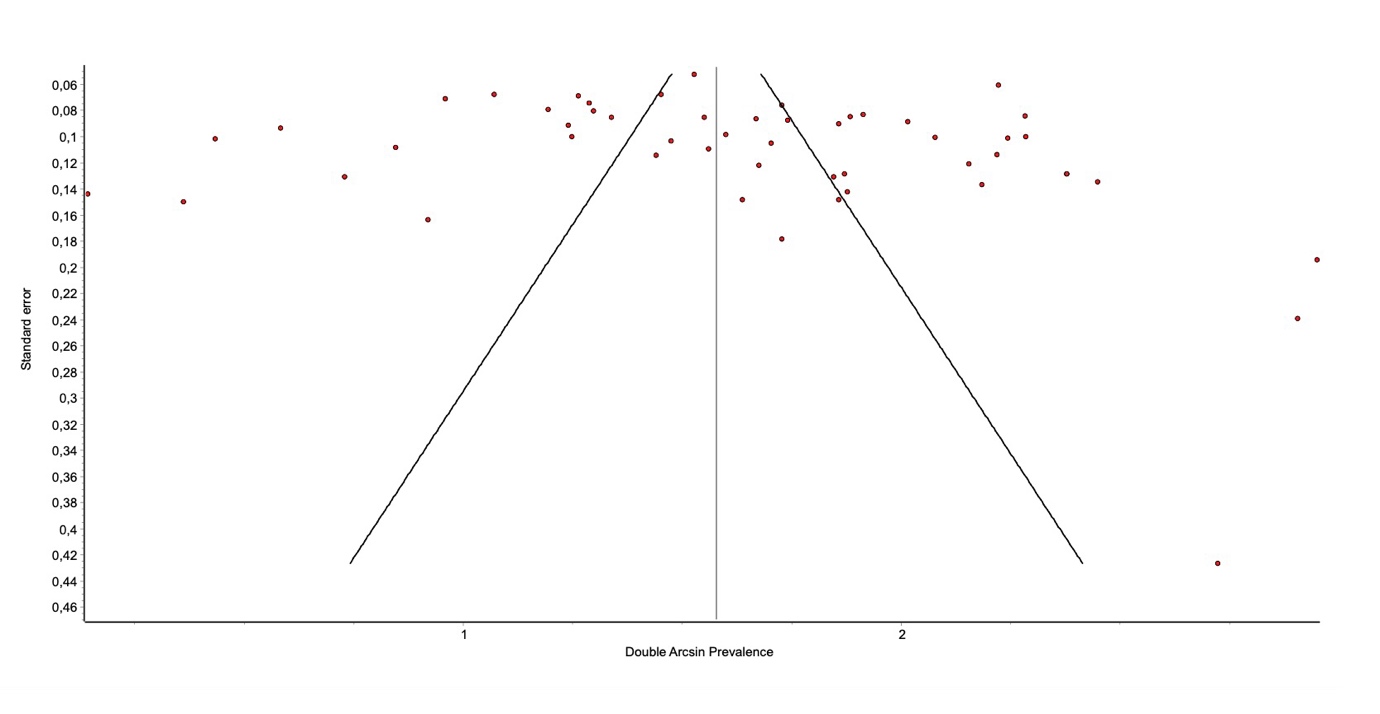


**Figure 3 Funnel plot showing overall publications on experimental infections of *Fasciola* species in their intermediate host snail.**


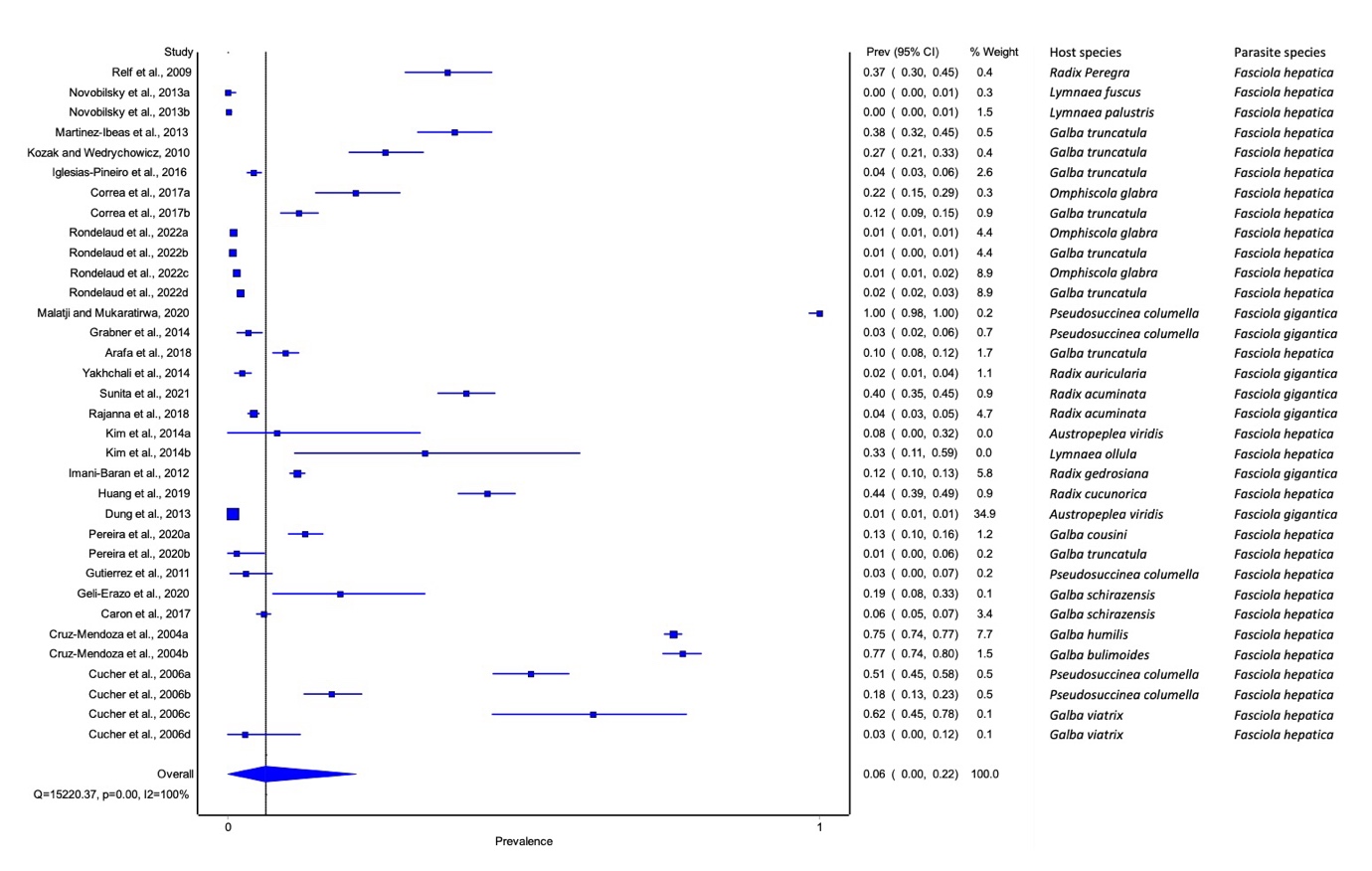


**Figure S4 Forest plot showing the overall rates of natural infections of lymnaeid snails with *Fasciola* spp. worldwide.**


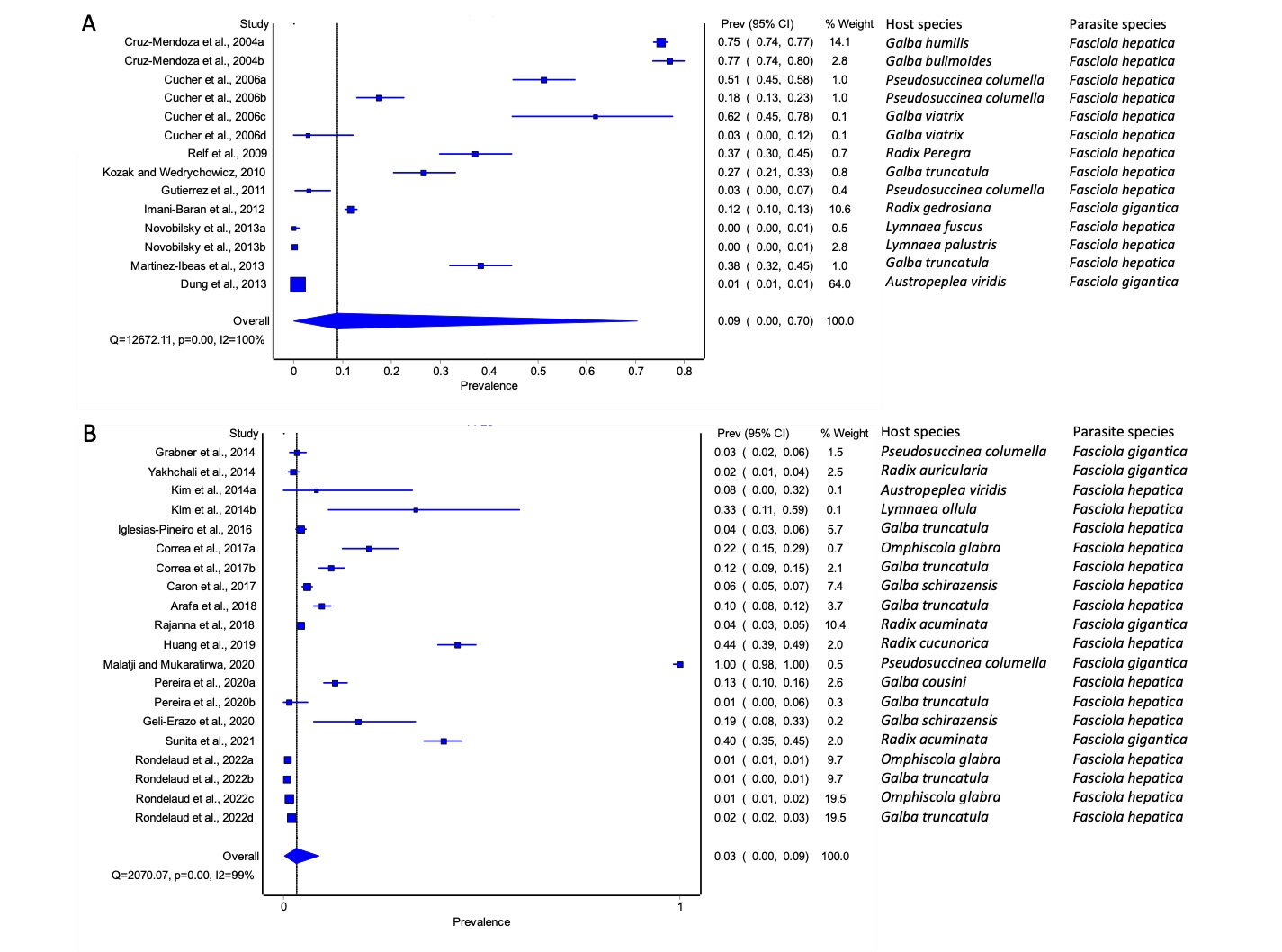


**Figure S5 Forest plots of the rates of infection of lymnaeid snails naturally infected with *Fasciola* species from (A) 2004-2013 and (B) 2014-2023.**


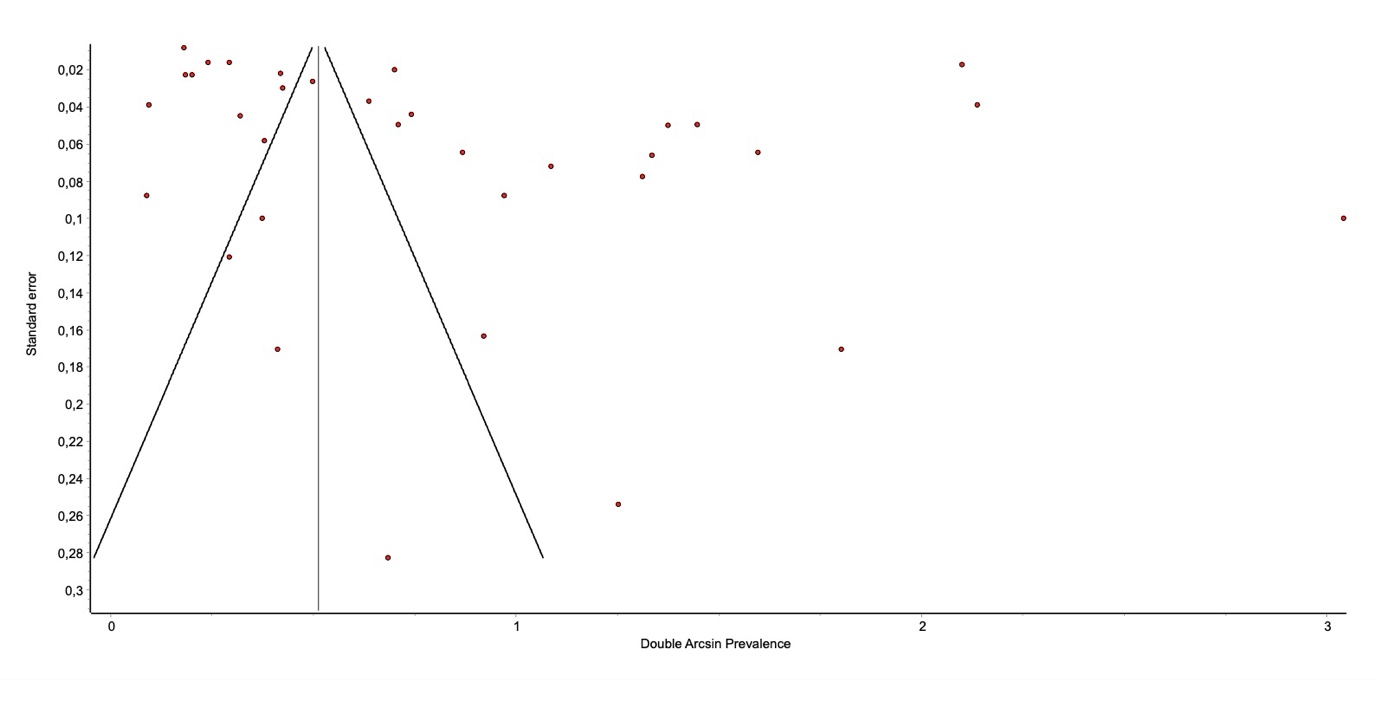


**Figure S6 Funnel plot of overall publications on natural *Fasciola* spp. on their intermediate snail hosts.**
