# Supplementary material for: Susceptibility of lymnaeid snails to Fasciola hepatica and Fasciola gigantica (Digenea: Fasciolidae): a systematic review and meta-analysis
Source: PeerJ. 2025 Mar 14;13:e18976. doi: 10.7717/peerj.18976 (PMC11913016; doi:10.7717/peerj.18976)
Supplement: Supplemental Information 3 [file peerj-13-18976-s003.docx]

**Table S1 Experimental infection of snail intermediate hosts with *Fasciola spp.* reported in the period 2004-2023.**

| **Continent** | **Country** | **Host species** | **Parasite species** | **Origin of strain** | **No. examined** | **No. infected** | **% infectivity** | **Detection technique** | **Index score** | **References** |
| --- | --- | --- | --- | --- | --- | --- | --- | --- | --- | --- |
| South America | Argentina | *Galba (G.) neotropica* | *Fasciola hepatica* | Argentina | 159 | 50 | 79.5 | Dissection | 5 | [43] |
| South America | Argentina | *G. truncatula* | *F. hepatica* | Argentina | 137 | 67 | 48.9 | Dissection | 5 | [43] |
| South America | Colombia | *G. cousini* | *F. hepatica* | Colombia | 100 | 34 | 34 | Dissection | 5 | [44] |
| South America | Colombia | *Pseudosuccinea (P.) columella* | *F. hepatica* | Colombia | 100 | 83 | 83 | Dissection | 5 | [44] |
| South America | Cuba | *P. columella* | *F. hepatica* | Cuba | 180 | 143 | 79.4 | Dissection | 5 | [45] |
| South America | Cuba | *G. cubensis* | *F. gigantica* | Cuba | 270 | 217 | 80.4 | Dissection | 5 | [46] |
| South America | Cuba | *P. columella* | *F. gigantica* | Cuba | 145 | 97 | 66.9 | Dissection | 5 | [46] |
| Africa | Egypt | *Radix (R.) natalensis* | *F. hepatica* | France | 263 | 62 | 23.6 | Shedding | 5 | [47] |
| Africa | Egypt | *R. natalensis* | *F. hepatica* | France | 45 | 24 | 53.3 | Dissection | 5 | [48] |
| Africa | Egypt | *P. columella* | *F. hepatica* | Egypt | 379 | 117 | 30.9 | Dissection | 5 | [49] |
| Africa | Egypt | *P. columella* | *F. hepatica* | France | 210 | 73 | 34.8 | Dissection | 5 | [50] |
| Africa | Egypt | *G. truncatula* | *F. hepatica* | France | 477 | 302 | 63.3 | Dissection | 5 | [50] |
| Africa | Egypt | *P. columella* | *F. hepatica* | France | 261 | 132 | 50.6 | Dissection | 5 | [51] |
| Africa | Egypt | *G. truncatula* | *F. hepatica* | France | 55 | 49 | 89.1 | Dissection | 5 | [51] |
| Africa | Egypt | *G. truncatula* | *F. hepatica* | France | 236 | 170 | 72.0 | Shedding | 5 | [52] |
| Africa | Egypt | *P. columella* | *F. hepatica* | France | 254 | 165 | 65 | Shedding | 5 | [52] |
| Europe | France | *G. truncatula* | *F. hepatica* | Egypt + France | 498 | 252 | 50.6 | Dissection | 5 | [53] |
| Europe | France | *P. columella* | *F. hepatica* | France | 319 | 104 | 32.6 | Shedding | 5 | [54] |
| Europe | France | *P. columella* | *F. hepatica* | France | 26 | 26 | 100 | Dissection | 4 | [55] |
| Europe | France | *G. truncatula* | *F. hepatica* | France | 77 | 62 | 80.5 | Dissection | 5 | [56] |
| Europe | France | *G. truncatula* | *F. gigantica* | Egypt | 83 | 41 | 49.4 | Dissection | 5 | [56] |
| Europe | France | *Omphiscola (O.) glabra* | *F. hepatica* | France | 103 | 53 | 51.5 | Dissection | 5 | [57] |
| Europe | France | *G. truncatula* | *F. hepatica* | France | 68 | 53 | 77.9 | Dissection | 5 | [57] |
| Europe | France | *G. truncatula* | *F. hepatica* | Argentina | 370 | 112 | 30.3 | Dissection | 5 | [58] |
| Asia | Iran | *R. auricularia* | *F. gigantica* | Iran | 294 | 115 | 39.1 | Shedding | 5 | [59] |
| Europe | Sweden | *G. truncatula* | *F. hepatica* | Sweden | 90 | 51 | 56.7 | Dissection | 5 | [60] |
| Europe | Sweden | *Lymnaea (L.) fuscus* | *F. hepatica* | Sweden | 114 | 9 | 7.9 | Dissection | 5 | [60] |
| Europe | Sweden | *L. palustris* | *F. hepatica* | Sweden | 119 | 40 | 33.6 | Dissection | 5 | [60] |

**Table S2** **Reports of naturally infected snail intermediate hosts with *Fasciola* spp*.* in the period 2004-2023.**

| **Continent** | **Country** | **Host species** | **Parasite species** | **No. examined** | **No. infected** | **Prevalence (%)** | **Detection technique** | **Index score** | **References** |
| --- | --- | --- | --- | --- | --- | --- | --- | --- | --- |
| Europe | Ireland | *R. peregra* | *F. hepatica* | 167 | 62 | 37 | Molecular | 5 | [61] |
| Europe | Sweden | *L. fuscus* | *F. hepatica* | 130 | 0 | 0 | Molecular | 5 | [60] |
| Europe | Sweden | *L. palustris* | *F. hepatica* | 668 | 1 | 0.15 | Molecular | 5 | [60] |
| Europe | Spain | *G. truncatula* | *F. hepatica* | 230 | 88 | 38.3 | Dissection | 5 | [62] |
| Europe | Poland | *G. truncatula* | *F. hepatica* | 192 | 51 | 26.6 | Molecular | 5 | [63] |
| Europe | Spain | *G. truncatula* | *F. hepatica* | 1141 | 50 | 4.38 | Dissection | 5 | [64] |
| Europe | France | *O. glabra* | *F. hepatica* | 130 | 28 | 21.5 | Molecular | 5 | [28] |
| Europe | France | *G. truncatula* | *F. hepatica* | 410 | 49 | 12 | Molecular | 5 | [28] |
| Europe | France | *O. glabra* | *F. hepatica* | 1950 | 19 | 0.9 | Dissection | 5 | [29] |
| Europe | France | *G. truncatula* | *F. hepatica* | 1948 | 16 | 0.6 | Dissection | 5 | [29] |
| Europe | France | *O. glabra* | *F. hepatica* | 3900 | 55 | 1.4 | Dissection | 5 | [29] |
| Europe | France | *G. truncatula* | *F. hepatica* | 3897 | 82 | 2.1 | Dissection | 5 | [29] |
| Africa | South Africa | *P. columella* | *Fasciola gigantica* | 100 | 100 | 100 | Molecular | 5 | [36] |
| Africa | Egypt | *P. columella* | *F. gigantica* | 296 | 10 | 3.38 | Molecular | 5 | [35] |
| Africa | Egypt | *G. truncatula* | *F. hepatica* | 731 | 71 | 9.7 | Molecular | 5 | [65] |
| Asia | Iran | *R. auricularia* | *F. gigantica* | 496 | 12 | 2.42 | RFLP | 5 | [66] |
| Asia | India | *R. acuminata* | *F. gigantica* | 400 | 161 | 40.25 | Molecular | 5 | [67] |
| Asia | India | *R. acuminata* | *F. gigantica* | 2077 | 89 | 4.3 | Molecular | 5 | [68] |
| Asia | South Korea | *Austropeplea (A.) viridis* | *F. hepatica* | 12 | 1 | 8.33 | Molecular | 4 | [21] |
| Asia | South Korea | *L. ollula* | *F. hepatica* | 15 | 5 | 42.7 | Molecular | 4 | [21] |
| Asia | Iran | *R. gedrosiana* | *F. gigantica* | 2543 | 298 | 11.72 | Molecular | 5 | [25] |
| Asia | China | *R. cucunorica* | *F. hepatica* | 409 | 179 | 43.76 | Molecular | 5 | [26] |
| Asia | Vietnam | *A. viridis* | *F. gigantica* | 15364 | 124 | 0.8 | Molecular | 5 | [27] |
| South America | Colombia | *G. cousini* | *F. hepatica* | 521 | 68 | 13 | Dissection | 5 | [69] |
| South America | Colombia | *G. truncatula* | *F. hepatica* | 68 | 1 | 1.4 | Dissection | 5 | [69] |
| South America | Cuba | *P. columella* | *F. hepatica* | 100 | 3 | 30 | Molecular | 5 | [19] |
| South America | Ecuador | *G. schirazensis* | *F. hepatica* | 37 | 7 | 19 | Molecular | 5 | [70] |
| South America | Ecuador | *G. schirazensis* | *F. hepatica* | 1480 | 89 | 6 | Molecular | 5 | [71] |
| South America | Argentina | *P. columella* | *F. hepatica* | 480 | 165 | 51.3 | Molecular | 5 | [22] |
| South America | Argentina | *G. viatrix* | *F. hepatica* | 68 | 22 | 61.8 | Molecular | 5 | [22] |
| North America | Mexico | *G. humilis* | *F. hepatica* | 3372 | 2537 | 75.2 | Dissection | 5 | [18] |
| North America | Mexico | *G. bulimoides* | *F. hepatica* | 670 | 515 | 76.9 | Dissection | 5 | [18] |
