# Supplementary material for: Susceptibility of lymnaeid snails to Fasciola hepatica and Fasciola gigantica (Digenea: Fasciolidae): a systematic review and meta-analysis
Source: PeerJ. 2025 Mar 14;13:e18976. doi: 10.7717/peerj.18976 (PMC11913016; doi:10.7717/peerj.18976)
Supplement: Supplemental Information 4 [file peerj-13-18976-s004.docx]

The audience the review is intended for

The review is intended for veterinary scientists, parasitologists, animal health specialists and policy makers from temperate and tropical areas, with a special focus on the epidemiology and control of trematodiasis, particularly fasciolosis. The review summarised Lymnaeidae and Racidinae species that are implicated in the transmission of specific *Fasciola* species in the field in various countries globally, and those that have been proven to potentially transmit *Fasciola* cercariae in the laboratory set-up. The review also provides data on the natural infections and prevalence of infections in snails, which is crucial in determining areas with active and continuous transmission of infection to definitive hosts. This information is critical in the case where various control strategies are implemented to control fasciolosis, including the interruption of the life-cycle at the larval stage.
